# TNS1: Emerging Insights into Its Domain Function, Biological Roles, and Tumors

**DOI:** 10.3390/biology11111571

**Published:** 2022-10-26

**Authors:** Zhihui Wang, Jingxue Ye, Fengrui Dong, Li Cao, Min Wang, Guibo Sun

**Affiliations:** 1 Institute of Medicinal Plant Development, Peking Union Medical College and Chinese Academy of Medical Sciences, Beijing 100193, China; 2 College of Pharmacy, Changchun University of Chinese Medicine, Changchun 130000, China

**Keywords:** tensin1 (TNS1), focal adhesion, cell migration, mechano-transduction, tumor

## Abstract

**Simple Summary:**

Tensin1 is a member of the tensin family, and tensins are adherent spot components. We summarize the role of tensin1 in various biological processes, such as cell adhesion, polarization, migration, invasion, proliferation, apoptosis, and mechano-transduction, through the available literature. Tensin1 is expressed in a variety of tissues, and the abnormal expression of tensin1 has been found in a variety of diseases; interestingly, we find that tensin1 plays a dual role in different types of tumors. We believe that these findings are important for future studies on the role of tensin1 in tumors.

**Abstract:**

Tensins are a family of cellular-adhesion constituents that have been extensively studied. They have instrumental roles in the pathogenesis of numerous diseases. The mammalian tensin family comprises four members: tensin1 (TNS1), tensin2, tensin3, and tensin4. Among them, TNS1 has recently received attention from researchers because of its structural properties. TNS1 engages in various biological processes, such as cell adhesion, polarization, migration, invasion, proliferation, apoptosis, and mechano-transduction, by interacting with various partner proteins. Moreover, the abnormal expression of TNS1 in vivo is associated with the development of various diseases, especially tumors. Interestingly, the role of TNS1 in different tumors is still controversial. Here, we systematically summarize three aspects of TNS1: the gene structure, the biological processes underlying its action, and the dual regulatory role of TNS1 in different tumors through different mechanisms, of which we provide the first overview.

## 1. Introduction

In 1991, tensin1 (TNS1) was identified as a component of focal contacts and other submembrane cytoskeletal structures that bind to actin. The SH2 domain of tensin binds to some proteins containing phosphotyrosine, and its tyrosine residues can be phosphorylated, suggesting that tensin may link signal transduction pathways to the cytoskeleton [1]. The mammalian tensin family includes four members. Tensin1 was the first identified one, and the other three members are tensin2 (TNS2), tensin3 (TNS3), and tensin4 (TNS4, also known as CTEN). The latter three share extensive similarities with TNS1, and recently, more research has been conducted on TNS1 than on the other three members [2].

TNS1 is a 220 kDa protein localized at the focal adhesion [3,4], which is a transmembrane linkage between the extracellular matrix (ECM) and the cytoskeleton [2]. TNS1 is a multidomain protein composed of conserved region 1 (C1), which is homologous to the protein kinase C; protein tyrosine phosphatase (PTP); C2; Src homology 2 (SH2); and phosphotyrosine-binding (PTB) domains [5]. Through these domains, TNS1 can anchor actin cytoskeletons and integrin receptors and transduce various types of signaling pathways through their binding partners; TNS1 is responsible for various cellular events, including cell adhesion, migration, polarization, proliferation [6,7,8], and apoptosis.

Studies on the expression of TNS1 in human tissues have revealed that the gene is localized to chromosome 2 and is highly expressed in the heart, kidneys, lungs, colon, prostate, testicles, and other organs [4]; contrastingly, the brain, thymus, and circulating leukocytes show low or no expression [3]. The expression of TNS1 is stimulated by angiotensin [9], oncogenes [10] (v-src, BCR-ABL), platelet-derived growth factor (PDGF) [11], and thrombin [12] and is suppressed by AMP-activated protein kinase [13]. The abnormal expression of TNS1 can cause numerous diseases by affecting downstream signaling pathways, such as the Rho GTP and PI3K/Akt/mTOR signaling pathways [14,15,16,17].

## 2. Structure

**Figure 1:**
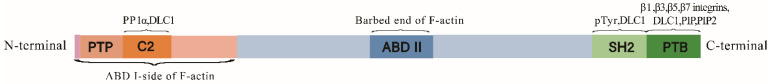
Domains of TNS1 and its binding partners.

TNS1 comprises different functional structural domains based on its characteristics as a focal adhesion molecule: the ABD I region proximal to the N-terminus of the focal adhesion-binding site (including PTP and C2 domains), the central ABD II region [18], and the C-terminus region of the focal adhesion-binding site consisting of two domains (SH2 and PTB). The biological functions of TNS1 depend on its structural domains and interacting proteins (Figure 1).

### 2.1. FAB (Focal Adhesion Binding)-N Terminal

#### 2.1.1. Protein Tyrosine Phosphatase (PTP)

Most PTP domains have the characteristic C-(X) 5-R motif; nevertheless, the PTP motif in TNS1 lacks key cysteine residues and is thought to be an inactive PTP [19].

#### 2.1.2. C2

In the tensin family, only the C2 structural domain of TNS1 contains a 299KVXF302 motif that binds to protein phosphatase 1α (PP1α) [15] and can recruit and bind serine/threonine PP1α to focal adhesions [20]. The C2 region drives TNS1 to interact with the sterile alpha motif (SAM) and Rho GTPase-activating protein (GAP) deleted in liver cancer 1 (DLC-1) to regulate RhoGAP activity in cells [14]. The PTP C2 structural domain, composed of ABD I, binds to the cytoplasmic side of cytoskeletal actin filaments [3].

### 2.2. ABD II: Non-Conserved Zone in the Middle

TNS1 contains the ABDII region, which is required to localize human TNS1 at cellular junctions, interact with actin filaments, and regulate their polymerization rate [3]. TNS1 is the only member of the Tensin family that has a structural domain [21].

### 2.3. FAB-C Terminal

The SH2-PTB structural domain is a unique part of the tensin family, whose pTyr motif is involved in signal transduction and the anchoring of local adhesions.

#### 2.3.1. Src Homology 2 (SH2)

The SH2 domain generally binds to proteins containing phosphorylated tyrosine residues, such as epidermal growth factor receptor (EGFR), focal adhesion kinase (FAK), phosphatidylinositol-3-kinase (PI3K), and p130 Crk-associated substrate (p130cas, also known as BCAR1). This interaction affects cell growth, polarization, migration, and other biological processes through protein tyrosine kinase-mediated signaling cascades [1,22,23,24]. However, the SH2 domain of TNS1 can bind to the non-phosphorylated tyrosine subunit of DLC-1 [25].

#### 2.3.2. Phosphotyrosine-Binding (PTB) Domain

The phosphotyrosine-binding (PTB) domain can bind with the NPxY motif of the β subunit of integrin to maintain its activity [26] in many cellular events, including cell adhesion, migration, and proliferation. Interestingly, the PTB structural domain of TNS1 can also bind to lipids. This binding pocket differs from the β-integrin recognition site [27], suggesting that both the SH2 and PTB structural domains of TNS1 are capable of interacting with lipids, unlike the predicted integrin β-tail recognition region. Therefore, lipid capture by the PTB structural domain of TNS1 may be important for protein function in focal adhesion.

## 3. Biological Processes in Which TNS1 Is Involved

In this section, we discuss the biological processes that are involved in TNS1 expression. TNS1 is a member of the adhesion complex and is involved in cell polarization, proliferation, apoptosis, and mechano-transduction [6,7,8], in addition to its commonly known involvement as a component of adhesive proteins in cell adhesion, invasion, and migration.

Tensin can serve as a platform for the dis/assembly of signal-transduction-related complexes in focal adhesion, because tensin can recruit tyrosine-phosphorylated proteins and provide binding sites for other SH2-containing proteins through its tyrosine phosphorylation [5].

### 3.1. Cell Adhesion, Polarization, Migration, and Invasion

#### 3.1.1. Cell Adhesion

The physical and signaling connections between cells, and between cells and the ECM depend on adhesion. Dynamic changes in cell adhesion affect processes, such as cell polarization, migration, proliferation, and mechano-transduction.

Surface properties such as wettability [28], a charge of the contact surface [29,30,31,32,33,34,35,36], and other factors can influence cell–matrix interactions. Because of the presence of phospholipids, proteins, and polysaccharide-coupling substances [37] with net negative charges on both the cell membrane surface and the ECM, the two repel each other and cannot come into direct contact but bind indirectly through cell membrane transmembrane receptors [38].

Integrins are transmembrane receptors that connect the cell membrane to the ECM (mainly fibronectin and laminin) and are heterodimers composed of α- and β-subunits [39,40]. The binding of integrin to the ECM induces ECM aggregation into adhesive patch complexes that mature to form adhesive patches, and finally, in the case of α5β1 integrin binding to fibronectin, fibrous adhesions [41]. These adhesion structures connect the ECM to the actin cytoskeleton, allowing the cells to generate forces that influence the extracellular environment [42].

An unknown focal adhesion-associated protein [43], which anchors cytoskeletal actin to adhesive patches to maintain the tension between the cytoskeleton and the plasma membrane, has been explored. This protein has been shown to cover the spiny ends of actin ribbons and pull actin filaments toward the cell membrane [44].

The unknown protein was initially thought to be vinculin because of its actin filament covering and binding activities [44]; however, after purification, these activities were lost. It was later shown that vinculin cannot cover the actin barbed ends, but the peptide co-stimulated with vinculin harbors end-capping activity [45]. Unexpectedly, Wilkins and Lin found that a low-purity vinculin protein preparation could recognize adherent spots. Further studies revealed that the low-purity protein preparation contained a spiky terminal capsid protein associated with focal adhesions, which is now known as tensin [46].

Up to this point, the identified structures associated with cell adhesion are dependent on focal adhesion to connect the ECM to the cytoskeleton, integrins as transmembrane receptors between the cell membrane and the ECM, and tensin between the cell membrane and actin filaments. What is the relationship between integrins and tensin?

Integrins themselves cannot bind directly to actin, and actin must mediate the activation and aggregation of integrins by binding to various adaptor proteins at specific sites in the tails of α- and β-integrins. Integrin adaptor proteins are broadly classified into three categories: those related to structure and function, catalytic activity, and binding sites for focal adhesion proteins. Tensin and talin, both belonging to the first category, are located in integrin-mediated cytosolic basement membrane junctions and can bind directly to cytoskeletal actin, thereby coupling integrins to the cytoskeleton and participating in cytoskeletal organization, cell migration, and proliferation [6,47]. Integrins are activated upon talin binding to its tail, facilitating binding to exocellular ligands (outside-in signaling) and inducing the recruitment of the cytoskeleton and signal protein network, collectively called the adhesome [48,49]. The composition of integrin-binding adaptors affects both the activation state of integrins and downstream signaling pathways; thus, adaptor proteins must be tightly regulated. Adaptor molecules change continuously during the transition from focal contacts to focal adhesion to fibrillar adhesion, and tensin is recruited from focal adhesion to fibrillar adhesion only when focal adhesion progresses to fibrillar adhesion. What are the specific changes in the molecular composition of the integrin junction during the maturation of adherent spots and the progression from adherent spots to fibrous adhesions?

As previously mentioned, both TNS1 and talin contain PTB domains that bind to the proximal NPxY motif of the membrane in the β-integrin tail [6]. Some PTB domains can only bind to tyrosine-phosphorylated NPxY, such as talin, which is unable to bind to phosphorylated tyrosine [50], and some are independent of phosphorylation status [51,52,53], such as tensins; their affinities for unphosphorylated and phosphorylated β1 (primarily localized to fibrillar adhesion) and β3 (primarily localized to focal adhesion) integrins are the same [54]. The phosphorylation of β-integrin at NPxY tyrosine residues affects the binding of TNS1 to talin, thereby acting as a regulatory switch. Therefore, what is the function of the talin-tensin1 phosphotyrosine switch? On the one hand, the adapter protein switches from talin to TNS1 to switch from structural adhesion to adhesion involved in signaling. Early in the formation of focal contacts, talin binds and activates β-integrins [55], and adapter proteins (e.g., talin and vinculin) are required between β-integrins and the cytoskeleton to reinforce the adhesion structure [56,57]. β-Integrins initially recruit talin, and once the focal contacts mature into focal adhesions, Src in the focal adhesions phosphorylates the β-integrin NPxY motif, thereby reducing the affinity between talin and β-integrin. Tensin1 increases its affinity for phosphorylated β-integrin and forms a signaling complex with PI3K or RhoGAP through its SH2 domain to activate Akt (via PI3K) and the recombinant actin cytoskeleton (via RhoGAP) to regulate downstream signaling pathways [6]. On the other hand, TNS1 binds to phosphorylated β-integrins, and that promotes its transfer into fibrillar adhesions, as it has been found that in Src-free cells, the phosphorylation level of focal adhesion is low and tensin1 is not contained in fibrillar adhesions [58]. The different affinities of tensin1 and talin as adapter proteins for phosphotyrosine are directly related to their different molecular structures. The PTB domain, which binds phosphotyrosine, usually contains a pocket containing basic residues that are partially coordinated with the phosphate moiety. TNS1 has two basic residues, Arg1704 and Lys1705, in the b6–b7 loop. The b6–b7 loop in the tensin1 PTB crystal structure is disordered, suggesting that it is mobile, which may contribute to the ability of this structural domain to adapt to phosphorylated or non-phosphorylated tyrosines, whereas the similar loop in talin is acidic [59], which is consistent with the fact that tyrosine phosphorylation disrupts integrin binding.

#### 3.1.2. Cell Polarization, Migration, and Invasion

Cell polarization is required for directed cell migration. Cells polarize, and the front of the cell extends pseudopods to form protrusions where new adhesions are formed, and the focal contacts mature into focal adhesions that transmit the traction required for movement [60]. Cell migration, also known as cell crawling, cell movement, and cell motility, refers to the movement of cells upon receiving migration signals and is an important biological process that underlies physiological and pathological processes, such as embryonic development, immune response, and tumor metastasis. Focal adhesions play an important role in cell adhesion, intracellular material transport, and cell migration through actin polymerization and contractile myosin linked to actin [61], which exerts traction on actin filaments and translates this force into cell motility via focal adhesion [62]. Cell invasion is the passage of cells across the ECM to another anatomically distinct tissue, and it is essential for tumor cells to metastasize to a normal tissue. During cell migration, dynamic alterations in the structure and composition of focal contacts may play key roles in cell attachment and detachment [3]. In a Boyden cell migration assay, 293 cells expressing tensin1 were detected to migrate at a faster rate than the 293 cells not expressing tensin, indicating that TNS1 promoted cell migration [63].

As we all know, focal adhesion kinase (FAK) plays an important role in cytoskeleton formation and cell migration; what is the relationship between FAK and TNS1? On the one hand, FAK phosphorylates the NPxY motif of β-integrin by forming the FAK/Src complex, increasing the binding of TNS1 to β-integrin, generating the assembly complex of TNS1 and PI3K, and promoting cell polarization and migration [6]; on the other hand, FAK/Src complex promotes cell migration by activating downstream p38MAPK and then phosphorylating the Ser/Thr site of TNS1, increasing its recruitment to FAK, p130Cas [64].

The different types of focal adhesions are usually classified according to their subcellular location, size, and composition as initial (or nascent) adhesions, focal complexes, focal adhesions, fibrillar adhesions, podocytes, and three-dimensional (3D) matrix adhesions [41,65]. In focal adhesions, intracytoplasmic adaptor proteins are mainly high levels of talin, paxillin, and highly phosphorylated tyrosine and are mainly associated with αvβ3 integrins, mainly distributed in the periphery. However, fibrillar adhesions contain α5β1 integrins and low levels of paxillin and vinculin and are largely devoid of phosphotyrosine. The most prominent cytoskeletal component of fibrillar adhesions is tensin [66]. These fibrillar adhesions emerge from focal contacts and translocate in an actin-dependent manner toward the cell center, forming linear or punctate arrays rich in tensin [67]. The mechanisms by which these transformations occur are as follows: Talin and tensin1 are both adapters of the PTB structural domain, and they both bind to the proximal NPxY motif of the membrane in the tail of the β-integrin. They are just recruited to the focal adhesions at different time points [68,69]. β-Integrins possess no enzymatic or actin-binding activity of their own [6]. Cell adhesion to the ECM leads to the aggregation of integrins and cytoskeletal proteins at the adhesion sites, and cytoskeletal proteins such as talin and paxillin linked to β-integrins recruit FAK to the adhesion sites [70,71,72], where FAK is activated by integrins and autophosphorylated at the Y397 Tyr site [73]. Activated and phosphorylated FAK leads to binding to several intracellular signaling molecules, such as Src, Grb2, and PI3K [74]. Src phosphorylates the NPxY motif by interacting with FAK and the β-integrin tail itself to localize focal adhesions [75,76,77], thereby weakening the interaction between talin and the β-integrin tail; instead, tensin may interact with the phosphorylated NPxY motif in an enhanced manner and assemble a signaling complex with PI3K and RhoGAP through its SH2 structural domain; this complex may regulate downstream signaling pathways through the activation of Akt (via PI3K) and the recombinant actin cytoskeleton (via RhoGAP) to regulate downstream signaling pathways [6].

In addition, FAK or the FAK/Src complex activates MAPK in two ways. In the first, the FAK/Src complex activates the downstream Grb2/Sos signaling pathway and subsequently the Ras/MAPK signaling pathway [78,79]. In the second, FAK and/or the FAK/Src complex phosphorylates the cytoskeletal protein paxillin in a manner similar to FAK tyrosine phosphorylation and p130Cas; the phosphorylation of paxillin [80] and p130Cas [81,82] leads to their binding to Crk and other receptor molecules, such as Nck, providing connections to possible downstream signaling pathways such as the MAP kinase pathway [74,83,84].

**Figure 2:**
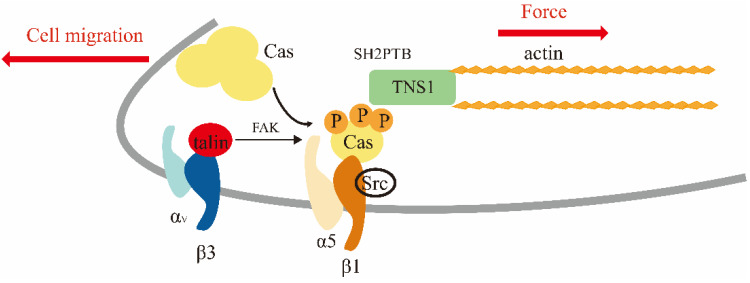
A model of the actin-Cas linkage.

As mentioned above, FAK/Src phosphorylates β-integrin, resulting in an increased binding of TNS1 to integrin, and in addition, the C-terminus of TNS1 has domains SH2 and PTB, which can interact with FAK and p130Cas [1,85]. Sorbitol activates p38 MAPK, which phosphorylates Ser/Thr of tensin1, and its SH2 binding properties are altered to recruit more pTyr site-containing proteins, including p130Cas and FAK [64]. Cas combines directly with FAK and Src [86,87]. Src tethers Cas molecules to the adhesion complex and phosphorylates tyrosine residues in their substrate structural domains, rather than simply Src binding to Cas. Phosphorylated Cas binds to TNS1 and constitutes the cytoskeleton adhesion linkage. A sustained inward flux of actin and phosphorylated CasSD promotes the inward displacement of Cas molecules from the adhesion complex of migrating cells and facilitates cell migration [88]. This mechanism is shown in Figure 2.

DLC-1 is a negative regulator of tumor formation and plays a role in cell migration [25,89,90]. DLC-1 contains three structural domains: N-terminal sterile alpha motif (SAM), Rho GAP, and C-terminal steroidogenic acute regulatory-related lipid transfer (START) domains [91]. A common feature of many tumors is the activation of Rho-GTPases, which are Ras-related proteins that may contribute to various parameters of abnormal cell growth, including viability, migration, invasion, and proliferation [92,93,94]. Rho GTPase is active when bound to GTP and inactive when bound to GDP. Rho-GDP can be changed to Rho-GTP via the stimulation of Rho-specific guanine nucleotide exchange factor activation, and Rho-GTP is hydrolyzed to Rho-GDP by Rho-specific GTPase-activating protein (RhoGAP) [90]. The three structural domains of TNS1, C-terminal SH2, PTB, and N-terminal C2, can all bind constitutively to different sequences of DLC-1, which are essential for the formation of focal adhesions and, more importantly, their interaction affects RhoGAP activity and are instrumental in cell polarization and migration. The binding of SH2 or PTB of TNS1 to DLC-1 does not affect the action of RhoGAP, resulting in decreased levels of RhoGTP and downstream ROCK, a reduced phosphorylation of MLC20, weakened actin contraction, and the inhibition of cell migration; in contrast, the binding of C2 of TNS1 to DLC-1-SAM suppresses RhoGAP activity and promotes cell migration [14,15,90,95].

Hall et al. found that in addition to RhoGAP-DLC-1, TNS1 also controls cell polarization, migration, and invasion through PP1α. The effect of TNS1 on cell polarization varies depending on the protein. The binding of Tensin1 to PP1α, on the one hand, promotes its binding to DLC-1 to inhibit cell migration, and on the other hand, it elevates MLC phosphatase activity in a DLC-1-independent manner, decreases MLC phosphorylation, decreases cell polarization, and decreases myosin contractility [15].

### 3.2. Mechano-Transduction

All cells are stimulated by external mechanical forces such as shear stress, stiffness, and tension [96,97]. Cells have evolved numerous mechanisms for sensing, processing, and linking intra- and extracellular signals to modify the intra- and extracellular environments in favor of their own evolution [98,99]. In this case, the mechanism that converts physical stimuli into biological or electrical signals is called mechano-transduction [100,101,102,103]. Mechano-transduction affects a wide range of cellular processes, such as cell proliferation, migration, and apoptosis [99,104].

The mechano-transduction process consists of mechano-sensing (the perception of mechanical stimuli), mechano-transmission (the transmission of stimulus signals to signaling events), and mechano-response (the functional response of the cell to mechanical stimuli). Mechano-sensing is the perception and propagation of mechanical cues at the cell surface dependent on plasma membrane receptors, their associated proteins, and the plasma membrane itself, triggering a signaling cascade that generates a mechanical response. Integrins, G protein-coupled receptors, enzyme-linked receptors (i.e., receptor tyrosine kinases), and ion channels can act as “mechanosensors” [105]. Different mechanosensors perceive mechanical stimuli to activate different mechano-transduction pathways that play significant roles in the progression of various diseases, especially tumors [106,107,108]. TNS1 and the various adhesion complexes it forms have been reported to be involved in mechano-transduction as mechanosensors [26,109].

The hippo pathway, discovered in *Drosophila melanogaster* about 20 years ago [110,111,112], controls organ size in animals by regulating cell proliferation and survival. yes-associated protein (YAP) and transcriptional coactivator with PDZ-binding motif (TAZ) are the core effectors of the hippo signaling pathway [113,114]. The Hpo network is primarily a core kinase cascade, involving Hpo/mammalian Ste20-like kinases 1/2 (MST1/2), Salvador (Sav)/SAV1, Mats/MOB kinase activator 1A/B (MOB1A/B), and Warts (Wts)/large tumor suppressor kinase 1/2 (LATS1/2). MST1/2 phosphorylates SAV1, MOB1A/B, and LATS kinases by forming a heterodimer with SAV1. LATS1/2 directly phosphorylates multiple sites of Yki’s immediate relatives YAP and TAZ, thereby inhibiting their nuclear localization [114]. Unlike conventional signaling pathways involving dedicated ligand-receptor pairs, Hippo-YAP signaling is subject to a wide range of architectural and mechanical cues, as well as biochemical signals including extracellular matrix stiffness, cell–cell adhesion, cell–matrix adhesion, cell density, cell shape, and cell polarity [115]. In non-muscle cells, actin consisting of F-actin and non-muscle myosinII is arranged in bundles, and RhoGTPase is a regulator of actin bundle actomyosin bundle assembly and function [116,117]. Contractile myosin and RhoGTPase act as central mediators between mechanical cues and actin-dependent Hippo-YAP signaling in various mechanically induced environments [113,116,118,119]. RhoGTPase perceives ECM stiffness through focal adhesions, which in turn promotes actin polymerization and stress fiber formation via the RhoA/ROCK downstream signaling pathway, and the high-intensity actin backbone inactivates the hippo pathway [119,120]. YAP/TAZ dephosphorylates and localizes to the nucleus [121]. These two proteins interact with members of the transcriptional enhancer factor (TEA)-domain (TEAD) binding factor family to regulate transcription. In vitro, under standard tissue culture conditions, most adherent cells display active YAP/TAZ, which is required for cell proliferation and migration [114]. Thus, the forced expression of YAP or TAZ in cells or tissues can effectively promote proliferation, lead to organ overgrowth, and almost invariably result in cancer [114,122]. In contrast, if the hippo signaling pathway is activated, YAP/TAZ is phosphorylated and retained in the cytoplasm and becomes inactive, limiting tissue growth and proliferation [123,124].

Unfortunately, although TNS1 is an focal complex, no studies have yet shown its direct involvement in the hippo pathway; instead, other focal complexes, such as the adaptor proteins Talin and Vinculin, link integrins to F-actin at the focal adhesions, affecting the nuclear localization of YAP/TAZ [125]. Nevertheless, TNS1 affects RhoGAP and RhoGTPase activity by interacting with DLC1 and PP1α. Therefore, TNS1 may regulate the hippo signaling pathway by affecting RhoGAP and regulates cell proliferation, which is important for the development of tumor.

**Figure 3:**
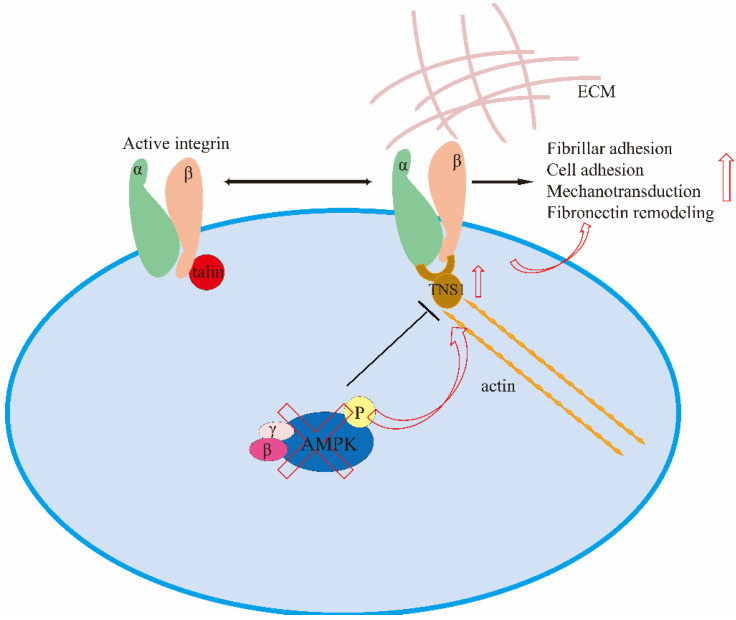
Mechanisms of AMPK-regulated fibrillar adhesion, cell adhesion, mechano-transduction, and fibronectin remodeling through the targeting of TNS1.

Both TNS1 and its adhesion complexes can act as mechanosensors that regulate various cellular physiological processes. If AMPK (an inhibitor of integrin β1) is lacking, TNS1 expression is promoted, and cells generate more mechanical stress with increased cell spreading, cell adhesion, fibrillar adhesion, and fibronectin remodeling [26]. This mechanism is shown in Figure 3. Meanwhile, in the past few years, it has been observed that the deprivation of glucose or the inhibition of glycolysis induces a strong inhibition of YAP/TAZ activity, at least in part, in an AMP-activated protein kinase (AMPK)-dependent manner [126,127,128,129]. AMPK appears to inhibit YAP/TAZ in multiple pathways, so it is speculated that YAP/TAZ may be involved in regulating the transcription of TNS1.

### 3.3. Cell Proliferation and Apoptosis

Satellite cells are dormant under physiological conditions. When muscle fibers are impaired, satellite cells are activated, proliferate, and mature into “myogenic cells,” which are then activated, proliferate, differentiate, and fuse to form new multinucleated myofibers. It was observed that the activation and proliferation of satellite cells and myogenic cells in TNS1 KO mice and the subsequent ability of myogenic cells to fuse into myofibers were diminished [130]. Zhou et al. found that transgelin/TNS1 signaling promoted CRC cell proliferation and invasion [131]. Shih et al. also found that endothelial cells isolated from TNS1 KO mice or those silenced with TNS1 siRNA showed a significant reduction in proliferation [14].

Sun et al. revealed that prazosin exerted a therapeutic effect on acute myeloid leukemia by downregulating TNS1 expression and inhibiting the phosphorylation of Akt and mTOR in the PI3K/Akt/mTOR pathway, which in turn inhibited cell proliferation and promotes apoptosis [17]. Additionally, prazosin treatment downregulates the expression of anti-apoptotic protein Bcl-2 and upregulates the expression of apoptotic proteins Bax and Caspase. The upregulation of TNS1 expression reverses the effects of prazosin in acute myeloid leukemia. Calpain regulates the downstream signaling pathways of integrin and cytoskeletal integrity. TNS1 is degraded by activated calpain II, which maintains cell morphology, and calpain inhibitors, which inhibit TNS1 degradation, lead to altered cell morphology, impaired cell motility, and focal adhesions [4].

The classical PI3K/Akt/mTOR signaling pathway is involved in cell proliferation and apoptosis. TNS1 promotes cell proliferation and inhibits apoptosis by regulating this pathway. Auger et al. showed that phosphatidylinositol 3 kinase (PI3K) activity in tensin immunoprecipitates from platelet-derived growth factor (PDGF)-treated cells and the binding of PI3K to tensin in anti-phosphotyrosine (AntipY) immunoprecipitates were transitory, and the amount of activity was greatly reduced, suggesting that the binding of the two is a biochemical mechanism. FAK is a potential bridge between PI3K and TNS1. The SH2 domain of TNS1 binds to phosphorylated tyrosine 925 of FAK, which has an optimal binding sequence for PI3K at tyrosine 950. Both TNS1 and FAK are recruited to the focal adhesion complex before the recruitment of the 85 kDa subunit of PI3K, and the recruitment of the 85 kDa subunit also depends on tyrosine kinase activity. Upon PDGF stimulation, the TNS1 and PI3K complexes formed with the help of FAK activate the PI3K/Akt/mTOR signaling pathway to promote cell proliferation and inhibit apoptosis [132].

## 4. TNS1 and Tumors

TNS1 is implicated in tumor development; however, the role of TNS1 in tumors is controversial.

### 4.1. The Role of TNS1 in Tumors

For tumors, the proliferation, migration, and invasion of cancer cells are crucial steps in cancer cell metastasis, and the impaired morphology of cancer cells leads to weaker cell adhesion and the promotion of cell spreading; therefore, proteins involved in actin cytoskeleton remodeling are key factors in enhancing cancer cell migration. Among the numerous types of adhesion proteins, tensin interacts with the intracellular environment and the ECM [13].

Tumors consist of tumor cells and various mesenchymal cells that constitute the tumor microenvironment and promote tumor progression. Most mesenchymal cells are tumor-associated fibroblasts (CAFs) expressing α-smooth muscle actin (α-SMA), which can be derived from fibroblasts, smooth muscle cells, endothelial cells, epithelial cells, bone-marrow-derived cells (e.g., mesenchymal stem cells (MSCs)) [133,134], and tumor-associated fibroblasts, and can promote organ fibrosis, tumor cell growth, infiltration, and migration [135].

**Figure 4:**
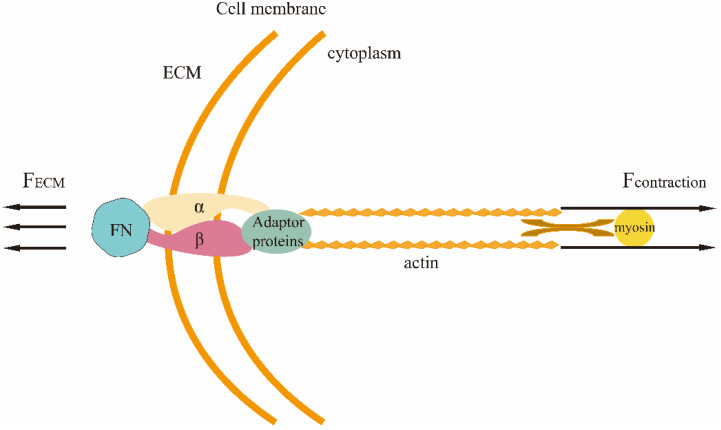
Tension homeostasis between cells and the ECM.

Changes in ECM rigidity lead to the differentiation of fibroblasts into CAFs. A mechanically relevant feedback mechanism exists within undifferentiated cells, where cells sense ECM stiffness through mechano-transduction and exert contractile forces at a magnitude proportional to this stiffness, thereby maintaining a dynamic force balance, called tensional homeostasis, between cells and the ECM through various types of adhesion-mediated mechano-transduction (Figure 4).

Under physiological conditions, ECM rigidity is normal, and cells proliferate normally [104,108]. If ECM stiffness is abnormally elevated (collagen deposition, fibrosis, etc.), the ECM resists myosin-mediated cell contraction; intracellular Rho-dependent cell contraction is enhanced in a compensatory fashion, and integrins aggregate [108,136,137]. This process can also be considered mechano-transduction [138], where Rho and ERK are activated [139], both of which together affect downstream ROCK and myosin activity [140,141], generating cytoskeletal tension and disrupting the dynamic balance of tension and driving malignant transformation [142]. ERK activation via integrin aggregation promotes cell proliferation [108].

**Figure 5:**
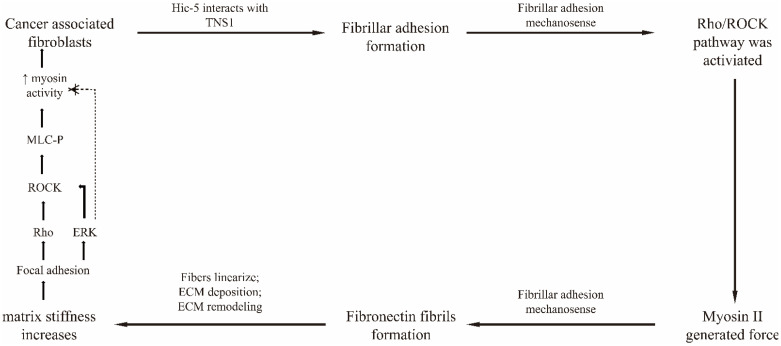
Mechanisms affecting tumor disease progression through mechano-transduction in tumor-associated fibroblasts.

Both Hic-5 (transforming growth factor β1i1) and TNS1 are mechanosensors, and the novel interaction between them is a mechanical signal for mechano-transduction [104,143,144]. Hic-5 is necessary for the formation of fibrillar adhesions in CAFs, and TNS1 is critical for the translocation of α5β1 integrins from focal adhesions to fibrillar adhesions. Increased matrix rigidity leads to Hic-5 (tyrosine phosphorylation), which mediates fibrillar adhesion in CAFs through direct interaction with the SH2 domain of TNS1. Mechanical signals are transmitted to the cytoskeleton via fibrillar adhesions via the Rho/ROCK signaling pathway. Myosin contraction generates a force on fibronectin [26,145], regulating fibronectin remodeling and fiber formation; directing the deposition of other mechanistic components [146,147]; reorganizing the ECM; changing matrix stiffness; and promoting tumor cell growth, invasion, and metastasis. The regulatory mechanism is illustrated in Figure 5.

As mentioned above, TNS1 may regulate the hippo signaling pathway by affecting RhoGAP and regulates cell proliferation and proliferation, which is important for the development of tumor. During tissue fibrosis, some inputs initiate the deposition of collagen whose stiffness activates YAP/TAZ. In turn, YAP/TAZ regulates the expression of cytoskeletal and ECM genes to maintain the activation and contractile state of fibroblasts and further promote ECM remodeling in a positive feedback loop [148,149,150]. Fibrosis is also a risk factor for many cancers. Indeed, cancer cells cooperate with cancer-associated fibroblasts to stiffen the tumor ECM, thus promoting tumor growth and metastasis [151]. Furthermore, the expression of typical EMT-inducible transcription factors such as Snail1/2, Slug, ZEB1 (Zinc Finger E-box-binding Homeobox 1), and Twist in various cancers is driven by YAP [152], suggesting that the hippo pathway is involved in the activation of the EMT program.

### 4.2. The Dual Role of TNS1 in Cancer

Although the role of TNS1 as a tumor suppressor has been widely demonstrated, in addition to the regulatory mechanisms described above and similar to those already mentioned, TNS1 can promote or inhibit tumor progression through the interaction of DLC-1 and PP1α. TNS1 has been shown to positively regulate tumor progression (Table 1).

#### 4.2.1. Colorectal Cancer

miRNA, a non-coding RNA of about 18–25 bp in length, can regulate gene expression [153]. LncRNAs, ~200 bp transcripts that cannot encode proteins, enhance the expression of target mRNAs via the phagocytosis of miRNAs [154,155,156].

The metastasis of cancer cells is an important cause of high mortality. Cancer metastasis is the process by which cancer cells spread from the primary site to distant sites to form tumors and is a complex multi-step process regulated by multiple factors [157,158].

TNS1 promotes colorectal cancer cell metastasis and tumor progression. In a comprehensive bioinformatics approach to identify differential genes unique to early-stage cancer patients, Singh et al. [159] conducted transcriptomic studies and revealed that TNS1 expression was upregulated in EOCRC samples, which may affect the MAPK signaling pathway along with other highly variable genes, thus promoting tumor cell migration and metastasis. TAGLN encodes transgelin, a 23 kDa actin-binding protein that promotes the proliferation and invasiveness of colorectal cancer cells by mediating TNS1 expression to promote pseudopod formation and cell motility, with low patient survival, the knockdown of TNS1 or TAGLN, and a reduced invasiveness of SW620 cells [131,160].

Nevertheless, several recent studies have found that TNS1 has two opposite functions in metastatic and primary colorectal cancers. Mi et al. found that miR-31-5p promotes colon adenocarcinoma (COAD) by repressing the target gene TNS1. TNS1 negatively impacts COAD tumorigenesis by enhancing M2 polarization, and the phosphorylation of TNS1 may accelerate metastasis by regulating epithelial-to-mesenchymal transition [161]. Zhang et al. found that the miR-31 expression level was higher in metastatic CRC than in primary CRC. TNS1, a downstream target gene of miR-31, was lower in primary tumor tissues than in normal tissues, but overall survival (OS) and disease-free survival (DFS) were improved in colorectal cancer patients with low TNS1 expression levels. Therefore, it is hypothesized that TNS1 regulated by miR-31 inhibits tumorigenesis and promotes the lymphatic metastasis of tumors [162].

#### 4.2.2. Gastric Cancer

Among all cancers, gastric cancer (GC) is the fifth leading cause of death [163]. Jiang et al. found that TNS1, an RNA-binding protein, was expressed at higher levels in peritoneal metastases than in the primary foci, and the proliferation of AGS (gastric cancer cells) cells was significantly decreased after TNS1 gene knockdown [164]. In addition, Marcin Nizioł et al. found a higher frequency of TNS1 in undifferentiated tumors than in hypo- and meso-differentiated tumors and high expression levels of TNS1 in metastatic tumors compared with those in tumors without distant metastasis [13]. These results suggest that TNS1 promotes the proliferation or metastasis of gastric cancer cells, positively regulating tumor progression.

#### 4.2.3. Breast Cancer

Breast cancer is one of the most common malignancies among women. Tumor cells transform into mesenchymal cells and spread outward to extend their sphere of influence, refine the surrounding ECM, and create a friendly environment for tumor cell growth [165,166,167,168].

It was found that the extracellular vesicles (EVs) isolated from metastatic breast cancer cells underwent EMT-MET (mesenchymal–epithelial transformation) and that TG2 (Transglutaminase-2) and FN expression was upregulated in the cells. Further studies revealed that TG2 promoted EMT and promoted metastasis by inducing EVs to produce TNS1; the deletion of TG2 or TNS1 significantly decreased EVs migration and did not affect primary tumor growth. Furthermore, Kaplan–Meier analyses showed that enhanced TNS1 expression was closely associated with reduced survival in patients with advanced breast cancer [63]. The knockdown of MATAR25 in highly aggressive 4T1 triple-negative (ER-, PR-, and HER2-) breast cancer cells resulted in a decrease in both cancer cell proliferation and metastasis. In addition, Kaplan–Meier survival analysis showed an increased expression level of TNS1 in breast tumors, further suggesting an active role of TNS1 in MaTAR25-mediated breast tumor progression. MaTAR25 is an lncRNA that functions as a molecular scaffold and/or chaperone, interacting with the transcriptional activator protein pur-beta (PURB) on the TNS1 gene promoter to promote TNS1 expression. TNS1 may promote cell motility and proliferation by mediating signal transduction between the extracellular matrix and actin cytoskeleton and participates in the EMT of cancer cells to promote breast cancer cell proliferation, migration, invasion, and metastasis [169].

These results suggest that TNS1 promotes the invasion and migration of breast cancer cells, but studies on different breast cancer cells by Zhan et al. yielded opposite results. miR-548j promoted breast cancer cell (MCF-7, SKBR3, and MDA-MB-231) invasion in vitro and metastasis in vivo (luciferase-tagged MDA-MB-231 cells were injected into NOD/SCID female mice via tail vein injection). The overexpression of miR-548j in breast cancer cell lines resulted in a significant decrease in endogenous Tensin1 levels; conversely, the inhibition of miR-548j resulted in the upregulation of Tensin1 expression. Breast cancer cells overexpressing TNS1 showed decreased invasiveness, and the knockdown of TNS1 in breast cancer cells increased invasiveness. The results of the in vivo experiments were consistent with the above, since Tensin1 was reported to interact with DLC-1, a member of the RhoGAP family, to regulate cell migration. Further studies revealed that CDC42-GTP accumulated upon miR-548j overexpression or TNS1 knockdown and that CDC42-GTP decreased upon miR-548j knockdown or TNS1 overexpression. siCDC42 or the selective CDC42 inhibitor ML141 inhibited the miR548j-mediated cell invasion pathway. In summary, miR-548j promotes breast cancer tumor invasion and metastasis but does not affect cell proliferation by inhibiting TNS1 expression, which in turn activates a Rho-GTPase—CDC42.

#### 4.2.4. Lung Cancer

MIR-31-5p was found to be significantly upregulated in lung adenocarcinoma (LUAD) tissues and cell lines H1299, H23, and A549, and TNS1, a direct target of miR-31-5p, was negatively correlated with the expression level of miR-31-5p. TNS1 expression was inhibited; TNS1-mediated activation of p53 was suppressed; and apoptosis was reduced [20]. Furthermore, TNS1 overexpression attenuates the pro-tumorigenic effect of miR-31-5p on LUAD cell function to some extent. In addition, LUAD patients with high TNS1 expression levels have a favorable prognosis, and TNS1 overexpression promotes p53 expression and apoptosis, which, to some extent, attenuates the pro-tumorigenic effect of miR-31-5p on LUAD cells [170,171].

#### 4.2.5. Bladder Cancer

In BCa tissues, lncRNA MAGI2-AS3, and TNS1 mRNA are expressed at low levels, and miR-31-5p is highly expressed, whereas in BCA patients, low expression levels of TNS1 are associated with tumor progression and poor prognosis. In a BCa cell line (T24 J82), by silencing lncRNA MAGI2-AS3, it was found that miR-31-5p expression was upregulated; TNS1 expression was downregulated; and the proliferation, migration, and invasiveness of BCa cells increased. In contrast, the overexpression of lncRNA MAGI2-AS3 and miR-31-5p expression were downregulated; TNS1 expression was upregulated; and the proliferation, migration, and invasiveness of BCa cells decreased. Finally, miR-31-5p overexpression promoted the proliferation of MAGI2-AS3-overexpressing BCA cells. These results suggest that MAGI2-AS3 promotes TNS1 expression by targeting the miR-31-5p/TNS1 axis to inhibit bladder cancer generation, proliferation, migration, and invasion [172].

#### 4.2.6. Renal Cancer

Martuszewska et al. have suggested that TNS1 may play a role in inhibiting renal cancer metastasis by disrupting the cytoskeletal structure. In addition, TNS1 expression is downregulated in human kidney tumors, and further studies have suggested that the upregulation of TNS1 expression may serve as an anti-cancer metastasis therapeutic strategy. For example, resveratrol significantly upregulates TNS1 expression in different cancer cells and exerts anticancer effects [173].

## 5. Conclusions

After years of research, we have achieved a better understanding of the structure, interacting proteins, biological functions, related diseases, and molecular regulation of TNS1. However, most such studies have not focused on the role of TNS1 in diseases, probably because TNS1 is expressed in multiple organs. Therefore, several questions remain to be answered. For instance, the contractile force generated by myosin is transmitted to the ECM through focal adhesions and affects the migration and invasion of cells; therefore, the continuous turnover of focal adhesions is crucial for cell migration. However, most of the current studies focus on the changes in adhesion molecules during the assembly of various types of adhesions and not on those during the disassembly of adhesion. According to existing research, TNS1 is closely related to tumors, but TNS1 is not a key oncogene; it only affects tumor progression, and there are no clinical drugs that directly target TNS1. Furthermore, it is not clear how it leads to tumor progression and whether it can be used as a marker gene for diagnosis and as a target for treatment. The role of TNS1 in tumors remains controversial and involves complex mechanistic pathways. Current findings include influencing RhoGAP activity and cell contractility through binding to DLC-1 and PP1α, affecting the EMT, and influencing apoptosis. Unfortunately, most experimental studies have not established a link to these mechanisms but have only explored the positive or negative effects of TNS1 on tumor progression, leaving the relevant mechanisms unclear. Nevertheless, it is interesting that TNS1 is involved in disease-related cell adhesion, proliferation, and apoptosis, and these insights may facilitate further research on the role of TNS1 in tumors. 

## Figures and Tables

**Table 1:** Positive and negative regulatory mechanisms of TNS1 in different tumors.

Tumor	Tumor Background	Positive Regulation	Negative Regulation
Colorectal cancer	Early-age-onset colorectal cancer Colorectal cancer Lymph node metastasis of colorectal cancer Colon adenocarcinoma	TNS1 affects MAPK signaling pathway and promotes cellular metastasis Pseudopod formation promotes cell proliferation and invasion TNS1 regulates cell movement to promote lymphatic metastasis of tumors Regulation of Hippo signaling TNS1 regulates EMT and accelerates tumor metastasis	TNS1 inhibits the production of the primary tumor TNS1 promotes Macrophage2 polarization and inhibits COAD tumorigenesis
Gastric cancer		TNS1 enhances cell contraction to promote proliferation and metastasis	
Breast cancer	Breast cancer extracellular vesicle (EV) fractions derived from metastatic breast cancer cells	TNS1 promotes the formation of focal adhesions; participates in EMT of cancer cells (4T1); and promotes proliferation, invasion, and migration of breast cancer cells Phosphorylation of TNS1 promotes EMT, promotes EV FN fiber formation, and promotes transfer	TNS1 inhibits CDC42 (Rho-GTPase) expression and inhibits cancer cells (MCF-7, SKBR3, MDA-MB-231) invasion and migration but does not affect proliferation
Lung cancer	Lung adenocarcinoma		TNS1 promotes p53 expression, promotes LUAD cell apoptosis, and inhibits LUAD cell proliferation, migration, and invasion
Bladder cancer			LncRNA MAGI2-AS3 promotes TNS1 expression by targeting miR-31-5p/TNS1 axis to inhibit bladder cancer generation, proliferation, migration, and invasion
Renal cancer			The mRNA level of TNS1 in RCCs negatively correlates with tumor grade

## Data Availability

This review does not present new experimental data. For data availability, please refer to the respective original papers.
